# Plastic bronchitis associated with adenovirus serotype 7 in children

**DOI:** 10.1186/s12887-020-02119-4

**Published:** 2020-06-03

**Authors:** Lin Yuan, Jing-jing Huang, Qi-guo Zhu, Ming-zhen Li, Zhi-qiang Zhuo

**Affiliations:** Department of Infectious Diseases, Xiamen Children’s Hospital (Children’s Hospital of Fudan University Xiamen Branch), 92 Yibin Road, Huli District, Xiamen City, 361006 Fujian Province China

**Keywords:** Adenovirus, Plastic bronchitis, Bronchoscopy, Children

## Abstract

**Background:**

Plastic bronchitis is an uncommon but severe respiratory disease characterized by formation of casts in tracheobronchial tree. It can lead to airway obstruction and even respiratory failure.

**Case presentation:**

Plastic bronchitis is mostly seen in both post-cardiac surgery patients, especially Fontan procedure, and infections including those caused by influenza viruses, Mycoplasma pneumoniae or tuberculosis. But it has rarely been reported to be associated with adenovirus infection. We report 2 cases of plastic bronchitis arising from adenovirus serotype 7 infection, manifested in repeated high fever, cough, and progressive dyspnea, and were diagnosed and eventually cured by bronchoscopy.

**Conclusions:**

Plastic bronchitis is a rare, variable and potentially fatal disease. In the cases we described, the cause was associated with adenovirus serotype 7 and its treatment required intervention with bronchoscopy and adequate control of the underlying disease.

## Background

Plastic bronchitis (PB) is a rare and underdiagnosed disease characterized by the formation and expectoration of bronchial casts, which can be potentially fatal [1]. Symptoms can range from cough and dyspnea to respiratory failure depending on the area of the compromised airway. Infection is one of the common causes of PB. The common pathogens reported are influenza virus (A and B), Mycoplasma pneumoniae (MP), EB virus, tuberculosis, fungus, etc. [2, 3]. Adenovirus is a common virus that causes community-acquired pneumonia in children, but there are few reports about PB associated with adenovirus infection, especially adenovirus serotype 7 [4]. We share our experience with two children who had PB associated with adenovirus serotype 7.

CARE guidelines N/A

## Case presentations

### Patient 1

**Fig. 1:**
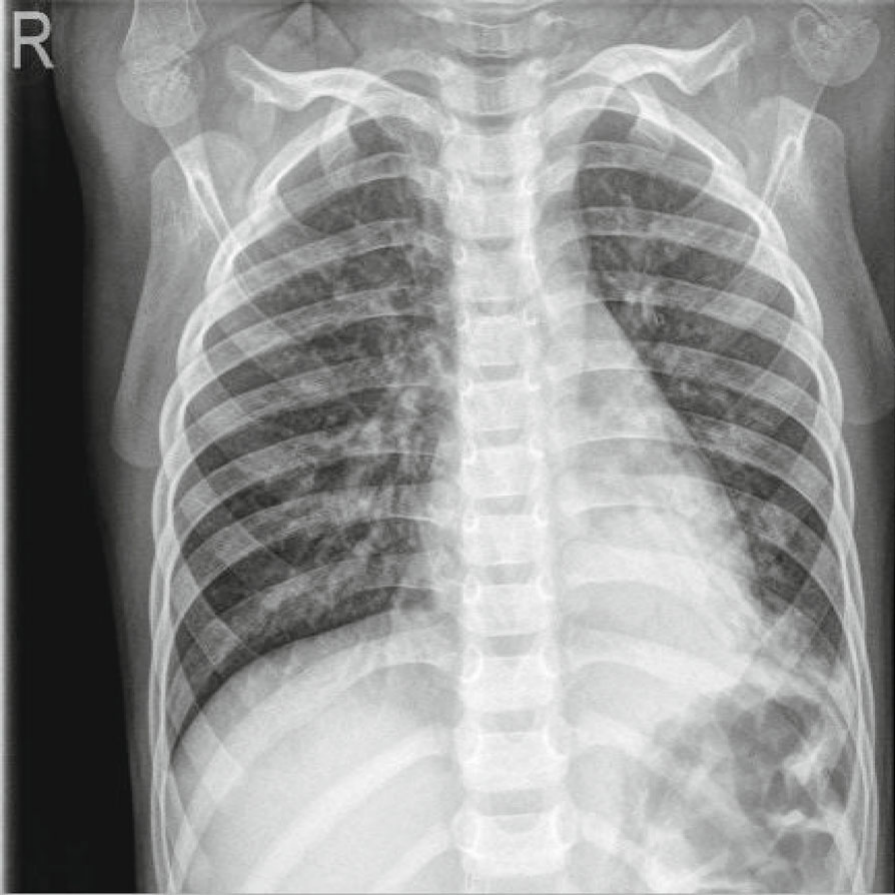
Chest X-ray of patient 1 at admission: atelectasis in the left lower lung

**Fig. 2:**
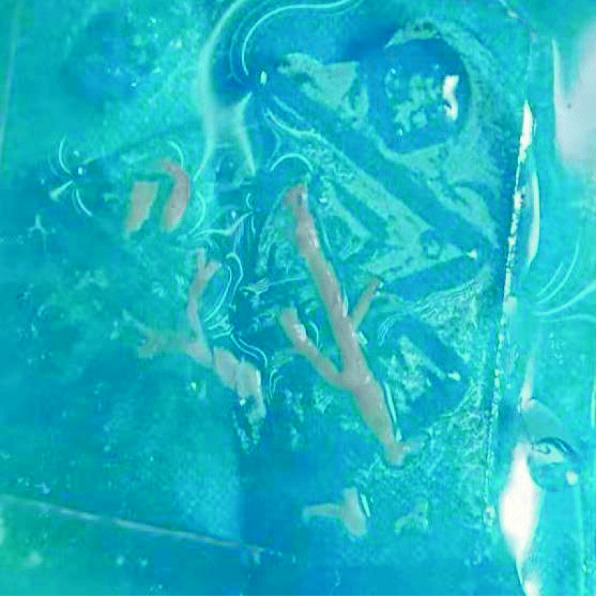
Cast removed from left lower lobe bronchus of patient 1

**Fig. 3:**
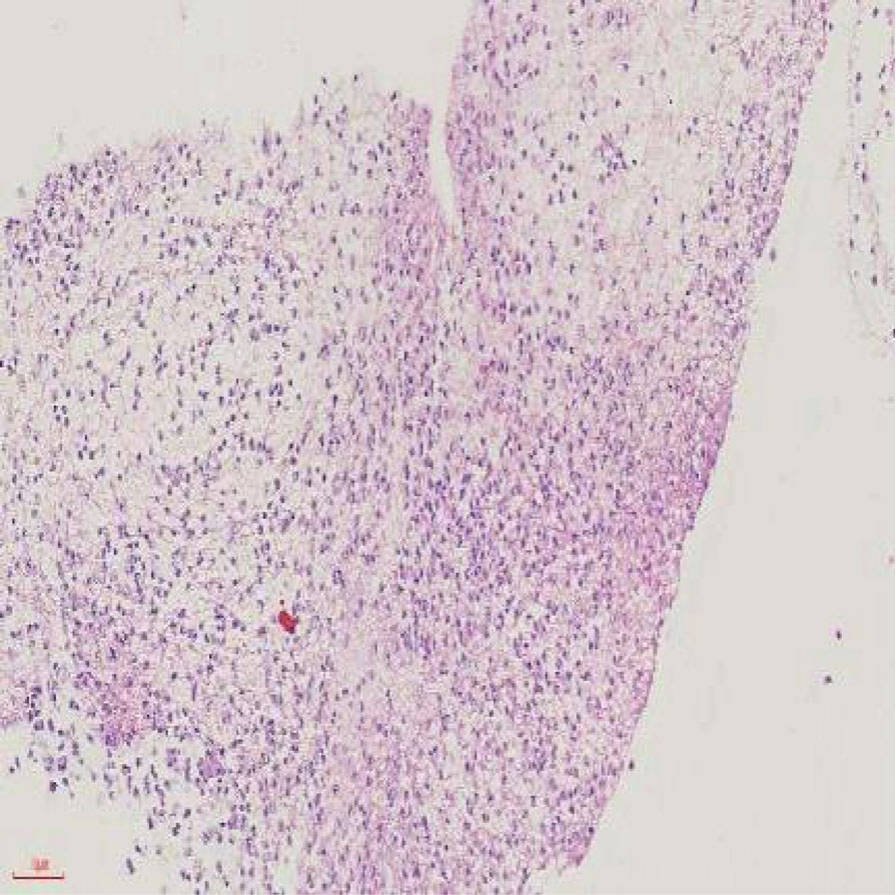
The cast was composed of inflammatory necrosis and neutrophils

**Fig. 4:**
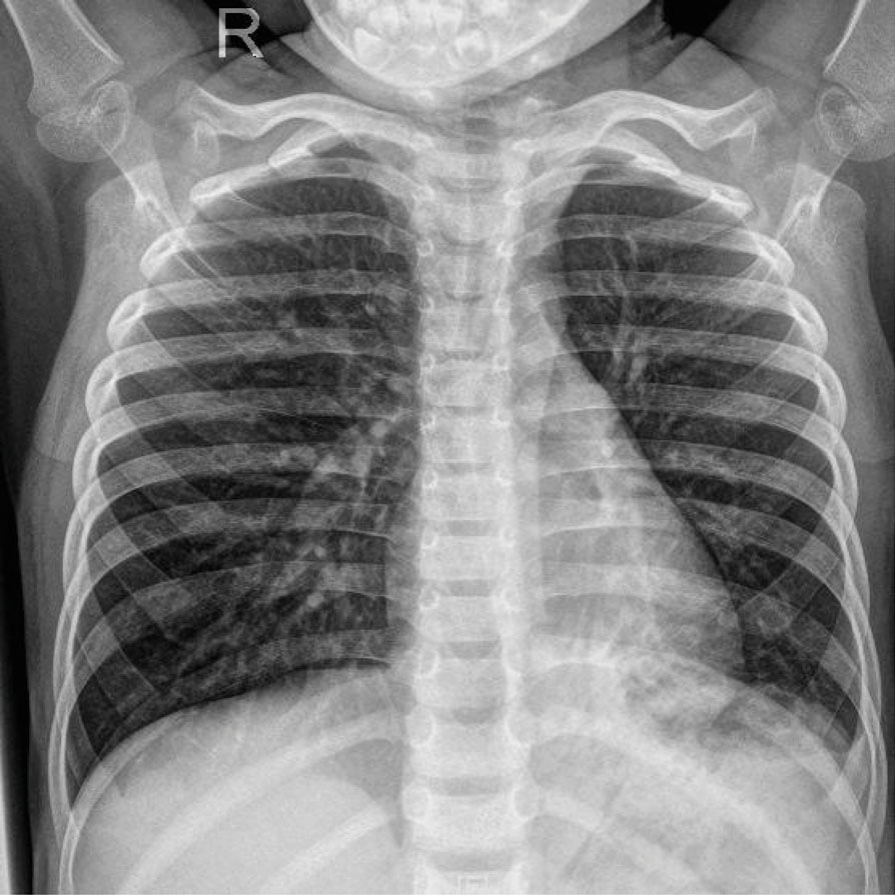
After treatment:the left lower lobe consolidation was significantly absorbed

A previously healthy 3-year-old girl was admitted to Xiamen Children’s Hospital after a 5-days history of fever and cough. At the local hospital, she was prescribed intravenous azithromycin for 2 days, but the temperature elevated again. On the day of admission, she had cough, fever (38.0 °C), shortness of breath, breath sounds of the left lower lung decreased, and we could hear moist rales and a little wheezing. The WBC count was 14.44 × 10^9^/L (normal ranges: (4–10) × 10^9^/L), her C-reactive protein level was normal, her procalcitonin level was elevated at 7.03 ng/ml, and lactate dehydrogenase was 743 U/L; A chest radiograph showed atelectasis of the left lower lung (Fig. 1). A chest CT scan showed segmental consolidation of the left lower lung and a small amount of effusion in the left thoracic cavity. Coagulation function shows elevated D-dimer and fibrinogen. Mycoplasma pneumoniae-IgM (MP-IgM) > 1:320. Nasopharyngeal swab was sent to the laboratory and Seven respiratory virus antigen tests (influenza A and B, parainfluenza 1, 2 and 3, respiratory syncytial virus and adenovirus) were negative. She received supplemental oxygen and antimicrobial treatment included azithromycin, and cefoperazone sulbactam sodium, all started immediately on admission. But her fever and cough persisted. On the 4th day of admission (the 9th day after the onset of the disease), we performed for her a flexible bronchoscopy, which revealed a whitish rubbery material occluding the left lower lobe bronchus, and plastic casts were removed (Fig. 2). The plastic casts were composed of inflammatory necrosis and neutrophils (Fig. 3). Genetic test for adenovirus serotype 7 in bronchial lavage fluid was positive. Two days after the bronchoscopy, the shortness of breath was improved, but the body temperature was still high. So we gave him gamma globulin (2 g/kg) to regulate immune function, on the 7th day of admission (the 12th day of the disease) her temperature was normal, coughing was alleviated, and discharged from the hospital 1 week later. One week after discharge, the chest radiograph showed that the left lower lobe consolidation was significantly better than before (Fig. 4).

### Patient 2

**Fig. 5:**
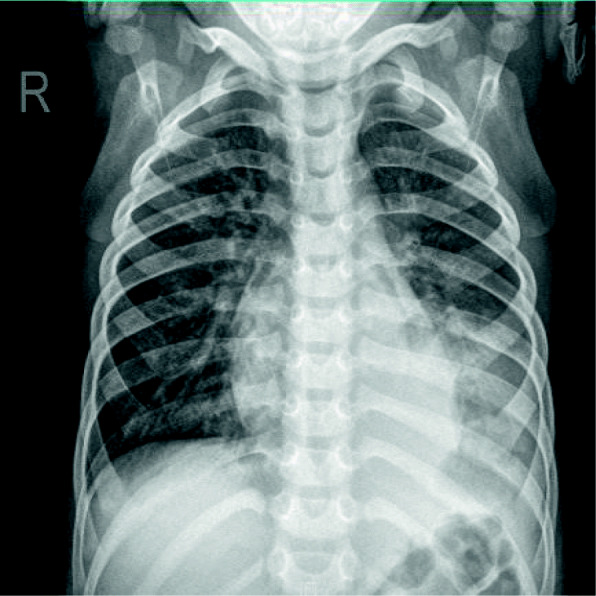
Chest X-ray of patient 2 atelectasis in the left lower lung

**Fig. 6:**
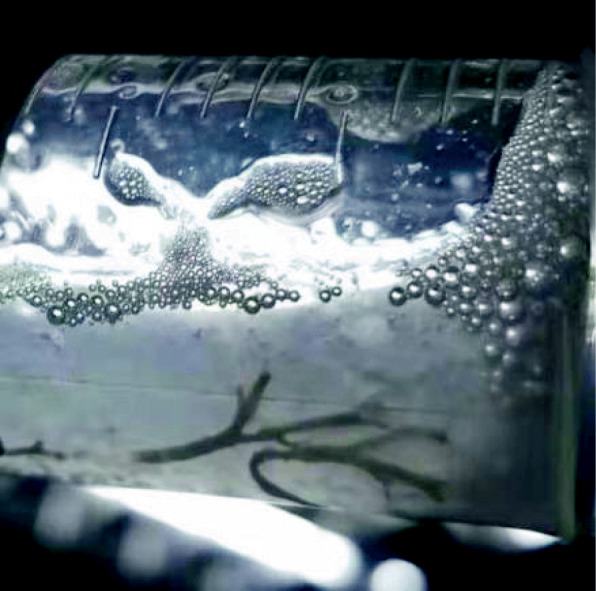
Cast removed from left inferior lobar bronchus of patient 2

**Fig. 7:**
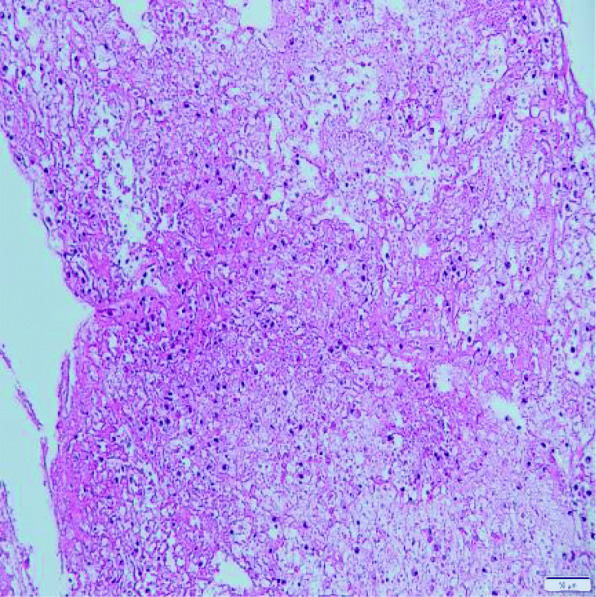
The cast was composed of a large quantity of inflammatory necrosis and neutrophils and a small number of lymphocytes

**Fig. 8:**
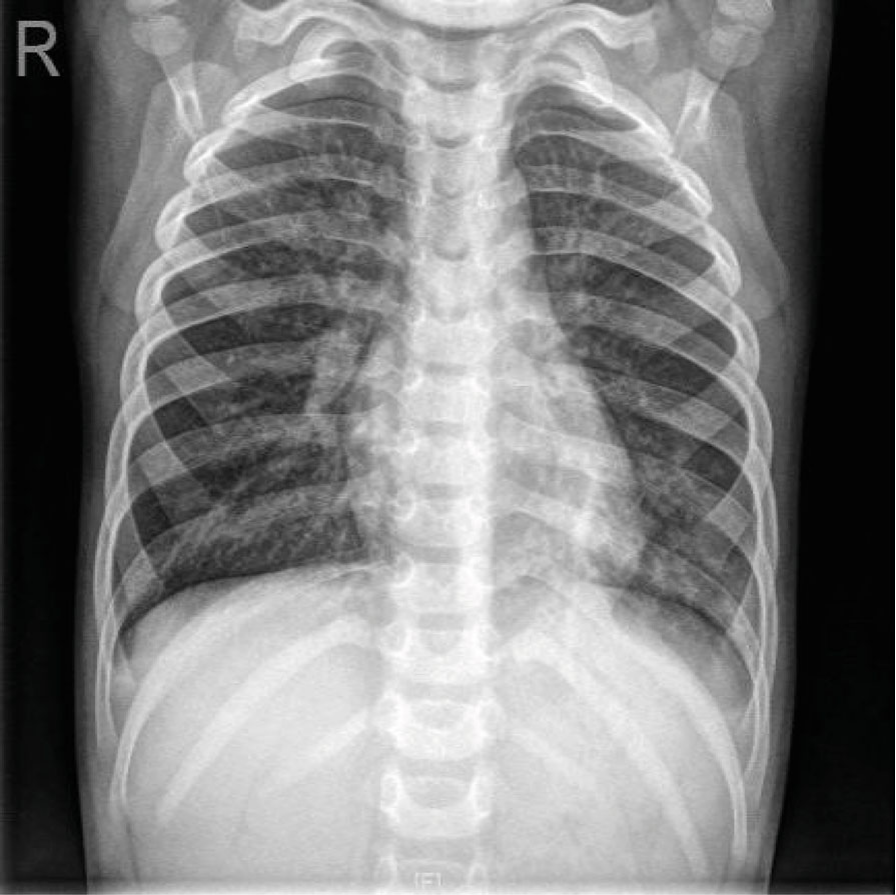
After bronchoscopy, a follow-up chest radiograph:the left lower lobe consolidation was significantly absorbed

A previously healthy 2-year-old boy was admitted to Xiamen Children’s Hospital because of a repeated fever for 1 month and cough for 20 days. At the local hospital, he was prescribed azithromycin, amoxicillin sulbactam against infection, but fever reccurred again after 5 days of stable temperature. On admission, the child was noted to have a normal breathing pattern and there were no auscultatory signs of note. His WBC count, C-reactive protein and procalcitonin level was normal, and lactate dehydrogenase was 602 U/L; bacterial cultures of blood and sputum were negative. The assay of specific IgM antibodies to seasonal influenza A and B, parainfluenza 1, 2 and 3, respiratory syncytial virus and adenovirus by enzyme immunoassay were all negative. MP-IgM also showed negative result; a chest X-ray showed both lungs were under-polarized, and the lungs were scattered with spots, patchy high-density shadows, and blurred borders. Antimicrobial treatment included ceftriaxone, and oseltamivir, but his temperature was still uncontrolled, and he gradually had difficulty breathing, his breath sounds over the left lower lung were decreased, and moist rales could be heard. The second chest radiograph showed that there was a atelectasis in the left lower lung (Fig. 5). And his C-reactive protein level was elevated (35.5 mg/L), procalcitonin level was elevated (3.71 ng/ml). Immediately, he received supplemental oxygen, cefoperazone sulbactam sodium for infection, gamma globulin (2 g/kg) to regulate immunotherapy and methylprednisolone (1 mg/kg twice daily) to inhibit inflammatory response. A couple of days later, despite subsistence of fever, shortness of breath persisted and a flexible fibre-optic bronchoscopy along with bronchial lavage, revealed a complete obstruction of the left inferior lobar bronchus by thick secretions (Fig. 6). Histologic examination of the plastic casts revealed a large quantity of inflammatory necrosis and neutrophils and a small number of lymphocytes (Fig. 7). Genetic test for adenovirus serotype 7 in bronchial lavage fluid was positive. After bronchoscopy, the shortness of breath was improved. A follow-up chest radiograph returned to normal (Fig. 8).

## Discussion and conclusions

Human adenovirus, which is non-enveloped, linear double-stranded deoxyribonucleic acid (DNA) virus, is a well known pathogen that cause a variety of human illnesses [5]. More than 50 distinct serotypes (such as 3, 5, 6, 7) have been identified since the early 1950s. Adenovirus serotype 7 can cause severe clinical presentation, a wide range of clinical syndrome and high mortality. PB associated with adenovirus serotype 7 is a severe and fatal acute lower respiratory illness that should be diagnosed and treated as soon as possible.

PB can occur in previously healthy children, although patients with allergy or asthma are at high risk of PB [6, 7]. Our patients had no history of atopy such as atopic dermatitis, allergic rhinitis and asthma. We reported 2 cases of PB associated with adenovirus serotype 7 who had acute onset, repeated fever and cough. Chest radiograph showed atelectasis. We finally diagnosed and cured PB through bronchoscopy. Removal of bronchial casts alone may not result in complete recovery and additional therapy involving gamma globulin infusions and systemic corticosteroids may be warranted.

There are two types of PB. Type I is associated with respratory diseases with inflammatory cells, fibrin and eosinophils. Type II is mainly associated with congenital heart disease and casts are consisted of mucus and a few cellular infiltrate [8]. We reported that pathological results of both cases indicated as type I, although one case infected by adenovirus serotype 7, another case infected by adenovirus serotype 7 and MP. We believe that PB induced by infection has a similar pathogenesis. At present it is believed that plastic casts is caused by a variety of inflammatory cell infiltrates and inflammatory mediators, resulting in tracheal mucosal congestion, edema, necrosis and blocking the lumen. There is no definitive conclusion as to which pathogen dominates the formation of PB. We believe that mixed infection has a synergistic effect on PB formation. In this study, the results of adenoviral antigen test for sputum or blood in 2 cases were negative. We believe that the main reason are both low sensitivity of specific antigen detection and improper sputum sampling. Adenoviral gene test of bronchial lavage fluid is beneficial for early diagnosis and treatment.

In conclusion, PB is a rare, variable and potentially fatal disease. In the cases we described, the cause was associated with adenovirus serotype 7 and its treatment required intervention with bronchoscopy and adequate control of the underlying disease.

## Data Availability

All data generated or analysed during this study are included in this published article.

## Abbreviations

PB: Plastic bronchitis
MP: Mycoplasma pneumoniae
